# NCAPG as a novel prognostic biomarker in numerous cancers: a meta-analysis and bioinformatics analysis

**DOI:** 10.18632/aging.204621

**Published:** 2023-03-29

**Authors:** Jie Lin, Gangyi Li, Yanping Bai, Yingjun Xie

**Affiliations:** 1 Department of Hepatobiliary and Pancreatic Surgery, Jilin University Second Hospital, Jilin 130000, China; 2 Department of Corneal Refraction, Jilin University Second Hospital, Jilin 130000, China

**Keywords:** NCAPG, tumor, meta-analysis, biomarker, bioinformatics analysis

## Abstract

Background: Identification of effective biomarkers for cancer prognosis is a primary research challenge. Recently, several studies have reported the relationship between NCAPG and the occurrence of various tumors. However, none have combined meta-analytical and bioinformatics approaches to systematically assess the role of NCAPG in cancer.

Methods: We searched four databases, namely, PubMed, Web of Science, Embase, and the Cochrane Library, for relevant articles published before April 30, 2022. The overall hazard ratio or odds ratio and 95% confidence intervals were calculated to assess the relationship between NCAPG expression and cancer survival prognosis or clinical characteristics. Furthermore, the aforementioned results were validated using the GEPIA2, Kaplan-Meier plotter, and PrognoScan databases.

Results: The meta-analysis included eight studies with 1096 samples. The results showed that upregulation of NCAPG was correlated with poorer overall survival (hazard ratio = 2.90, 95% confidence interval = 2.06–4.10, *P* < 0.001) in the cancers included in the study. Subgroup analysis showed that in some cancers, upregulation of NCAPG was correlated with age, distant metastasis, lymph node metastasis, TNM stage, relapse, differentiation, clinical stage, and vascular invasion. These results were validated using the GEPIA2, UALCAN, and PrognoScan databases. We also explored the processes of NCAPG methylation and phosphorylation.

Conclusion: Dysregulated NCAPG expression is associated with the clinical prognostic and pathological features of various cancers. Therefore, NCAPG can serve as a human cancer therapeutic target and a new potential prognostic biomarker.

## INTRODUCTION

Since the turn of the century, the incidence and mortality of cancer have increased significantly compared to that in previous years [1]. Although various treatment options, such as surgery, radiation, and chemotherapy, are available, the overall survival rates of patients with cancer have not improved significantly [2]. Therefore, identification of new tumor biomarkers and therapeutic targets can better guide clinical tumor treatment.

Non-SMC condensin I complex subunit G (NCAPG) is a subunit of the condensin complex, which condenses and stabilizes chromosomes during mitosis and meiosis [3]. Knockdown of NCAPG significantly reduced the viability of hepatocellular carcinoma cells by regulating Bax, cleaved caspase-3, E-cadherin, N-cadherin, cyclin A1, CDK2, and Bcl-2, and the expression of HOXB9 induces apoptosis and cell cycle arrest in the DNA synthesis phase [4]. In addition, upregulation of NCAPG can activate multiple signaling pathways to promote cell proliferation and anti-apoptotic activity and regulate DNA replication and mismatch repair in different cancer types [5–7].

Studies have shown that NCAPG is overexpressed in several tumors and associated with clinical features of cancer, such as tumor proliferation, metastasis, invasion, and patient survival [5, 8–10]. However, its role in various types of tumors remains controversial. For example, NCAPG is overexpressed in hepatocellular carcinoma [11] and glioma [12] but underexpressed in out-of-niche primary tumor cells of multiple myeloma and acute myeloid leukemia [13, 14]. Therefore, we performed a meta-analysis to explore the relationship between NCAPG upregulation and the clinical characteristics of cancer, analyze the prognostic value of NCAPG for cancer patients, and validate its role by bioinformatics methods.

## MATERIALS AND METHODS

### Literature search

Two authors independently searched four databases, namely, Pubmed, Embase, Web of Science, and the Cochrane Library for studies published before April 30, 2022. The following search terms were used: (“Neoplasms” OR “Carcinoma” OR “Prognosis” OR “Diagnosis” OR “Survival”) AND (“non-SMC condensin I complex subunit G” OR “NCAPG”).

### Inclusion criteria

The following inclusion criteria were considered when screening the databases: (1) the original literature was in English; (2) cancers with abnormal NCAPG expression were investigated; (3) high and low NCAPG expression was delineated; and (4) HR and 95% CIs of the OS can be obtained or calculated from the survival curve.

### Exclusion criteria

The exclusion criteria were as follows: (1) reviews, publication letters, retracted literature, and case reports; (2) insufficient data; (3) bioinformatics analysis; and (4) studies not relevant to NCAPG.

### Data extraction

Data extraction was performed by two investigators for all included studies and submitted to a third researcher to resolve disagreements. The following data were extracted according to the inclusion criteria: first author, publication date, country of origin, cancer type, number of cases, follow-up time, measurement method of NCAPG expression, outcome measures, HR and 95% CIs for OS.

### Quality assessment

The quality of the literature was evaluated using the Newcastle–Ottawa Scale. The evaluation was conducted independently by two investigators, and when disagreements arose, a third investigator participated in the discussion. The total score was 9 points, and a score of ≥6 points indicated high-quality research [15].

### Validation of the bioinformatics database

The GEPIA2.0 database (http://gepia.cancer-pku.cn/index.html) is a platform for sequencing and expression data, that includes most tumors and normal tissue samples [16]. Moreover, this study used the “Expression DIY” module to explore the differences between NCAPG transcripts from cancer tissue samples and normal tissue samples. In addition, we downloaded tumor transcription samples from the TCGA database (https://portal.gdc.cancer.gov/) [17] and used R (survival and timeROC packages) to perform a cox regression and ROC analysis of the survival rate. Next, we used the Kaplan-Meier Plotter database (https://kmplot.com/analysis/) to analyze the effect of the NCAPG gene on the survival rate in different cancers for additional data supplementation [18]. To validate the prognostic tumor status of NCAPG, we utilized the PrognoScan database to verify the survival information of this gene in multiple cancer datasets. We used the UALCAN database (http://ualcan.path.uab.edu/index.html) [19] to validate clinical information on NCAPG expression in tumors and explore the methylation and phosphorylation of NCAPG in these tumors.

### Molecular role and functional enrichment analysis of NCAPG

GeneMANIA (http://www.genemania.org) is an online tool for protein-protein interactions that helped in identifying genes with similar functions relative to that of the NCAPG gene in this study [20, 21]. We used the STRING database (https://string-db.org/) for GO and KEGG pathway analyses of NCAPG [22]. Finally, we used the multiMiR package (version 4.12) to identify competitive endogenous RNA of NCAPG and constructed a NCAPG network of competitive endogenous RNA interactions of target genes through Cytoscape software (version 3.8.2, https://cytoscape.org/) [23].

### Data processing and statistical analysis

The K-M curves of the included studies were processed by Enguage Digitizer 11.3 software to obtain HR values and 95% CIs. Further data analysis was performed using Review Manager 5.3 software. Survival outcomes were calculated by logarithmic HR values and their standard errors. In addition, the correlation between NCAPG upregulation and clinicopathological parameters of cancers (age, gender, degree of differentiation, TNM stage, metastasis, vascular invasion) was assessed by calculating the ORs and 95% CIs. Cochran’s *Q* test and I^2^ test assessed the heterogeneity to determine the effect model. A fixed model was used if the included studies had no significant heterogeneity (I^2^ < 50%, *P* > 0.1), while a random model was used otherwise. A sensitivity analysis of the included studies was performed using STATA 12.0 software to assess the stability of the results. Publication bias was evaluated using Begg’s rank correlation and Egger’s linear regression. At *P* < 0.05, publication bias was observed. If publication bias was present, then the trim-and-fill method was used to further assess the stability of the pooled results.

### Data availability statement

All relevant data is contained within the article: The original contributions presented in the study are included in the article, further inquiries can be directed to the corresponding authors.

## RESULTS

### Literature selection

**Figure 1:**
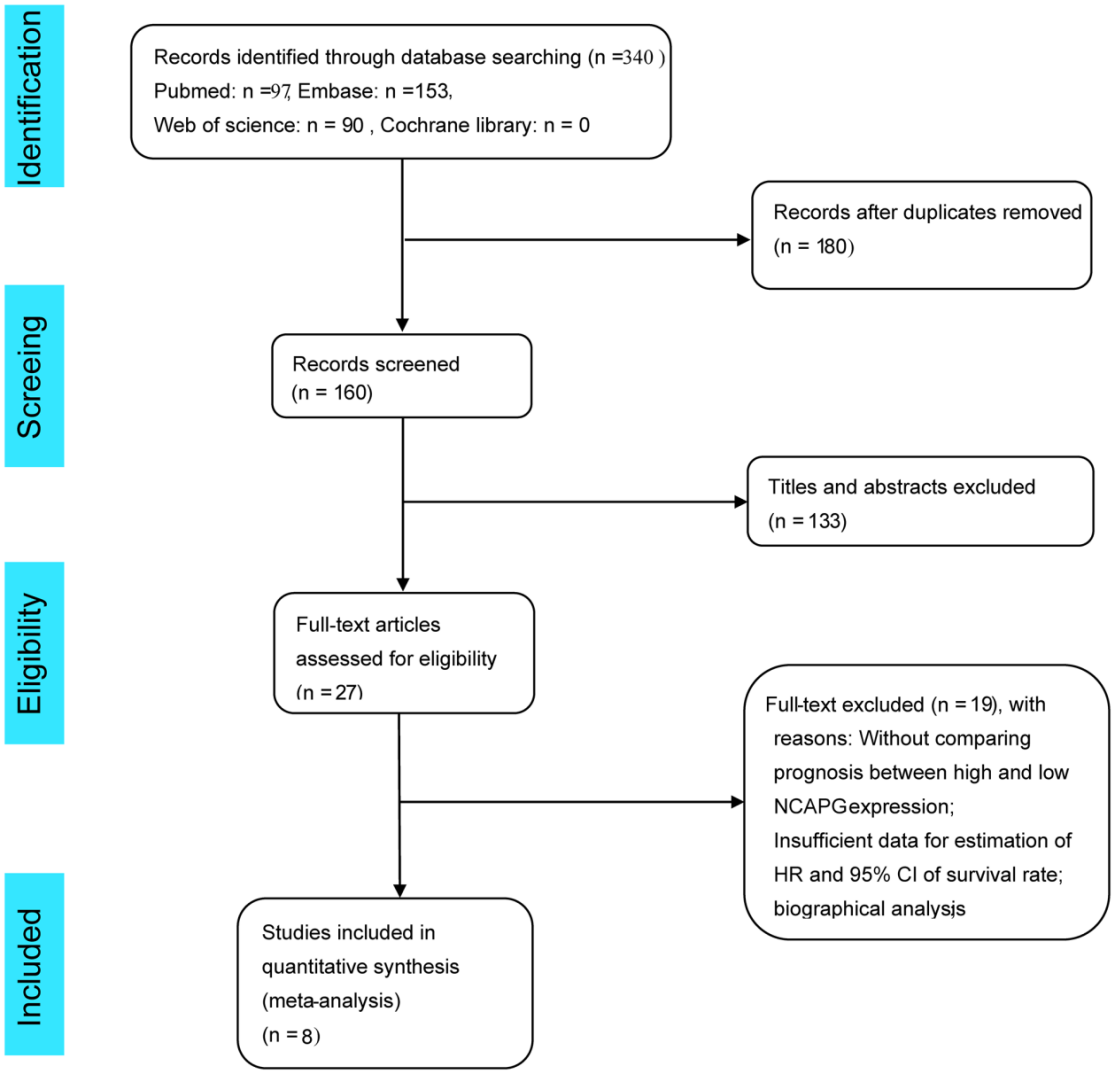
Flow chart of literature screening for this meta-analysis.

A flow chart depicting the literature screening process is shown in Figure 1. A total of 340 articles were screened, and 180 duplicate articles were excluded by searching PubMed, EMBASE, the Cochrane Library, and Web of Science. After scrutinizing the titles and abstracts, 133 more studies were excluded. As eight studies did not record the hazard ratios (HRs) and/or Kaplan-Meier (K-M) curves for overall survival (OS), 11 articles were excluded from bioinformatics analysis. Finally, we included eight studies in the meta-analysis [5, 6, 8, 9, 12, 24–26], all of which were cohort studies, and all outcome measures were OS.

### Study characteristics and quality assessment

The characteristics of the included studies are presented in Table 1. All the studies were conducted in China, and 1096 patients were recruited. The types of cancer included non-small cell lung cancer (NSCLC), glioma, breast cancer, gastric cancer, and hepatocellular carcinoma. All included studies assessed the association between OS and NCAPG expression, with follow-ups ranging from 80 to 140 months. All but one study reported clinicopathological parameters. All studies measured NCAPG expression via immunohistochemical staining, except for one study that performed RNA sequencing instead. Newcastle–Ottawa Scale scores were ≥6, indicating that the included studies were of moderate to high quality.

**Table 1:** Characteristics of included studies.

**Name**	**Year**	**Region**	**Ethnic**	**Type**	**Sample size (high/low)**	**FollowUp (months)**	**Method**	**Outcome**	**HR estimation method**	**HR (95% CI)**	**NOS**
Jiang	2020	China	Asian	BC	103 (35/68)	120	IHC	OS	K-M	7.57 (3.13, 18.29)	7
Sun	2022	China	Asian	NSCLC	156 (84/72)	140	IHC	OS, CP	REP	2.35 (1.3, 4.27)	7
Sun	2020	China	Asian	GC	135 (71/64)	120	IHC	OS, CP	REP	2.03 (1.23, 3.35)	7
Wang	2022	China	Asian	NSCLC	60 (28/32)	80	IHC	OS, CP	K-M	3.45 (1.26, 9.49)	6
Wang	2019	China	Asian	HCC	70 (35/35)	120	RNA-Seq	OS, CP	K-M	2.34 (1.08, 5.07)	7
Wu	2021	China	Asian	NSCLC	292 (164/128)	120	IHC	OS, CP	REP	2.05 (1.35, 3.11)	7
Zheng	2022	China	Asian	Glioma	140 (47/93)	120	IHC	OS, CP	K-M	9.34 (3.75, 23.26)	7
Zhou	2022	China	Asian	NSCLC	140 (70/70)	100	IHC	OS, CP	REP	2.32 (1.41, 3.81)	6

### Correlation of NCAPG expression with OS and cancer type

**Figure 2:**
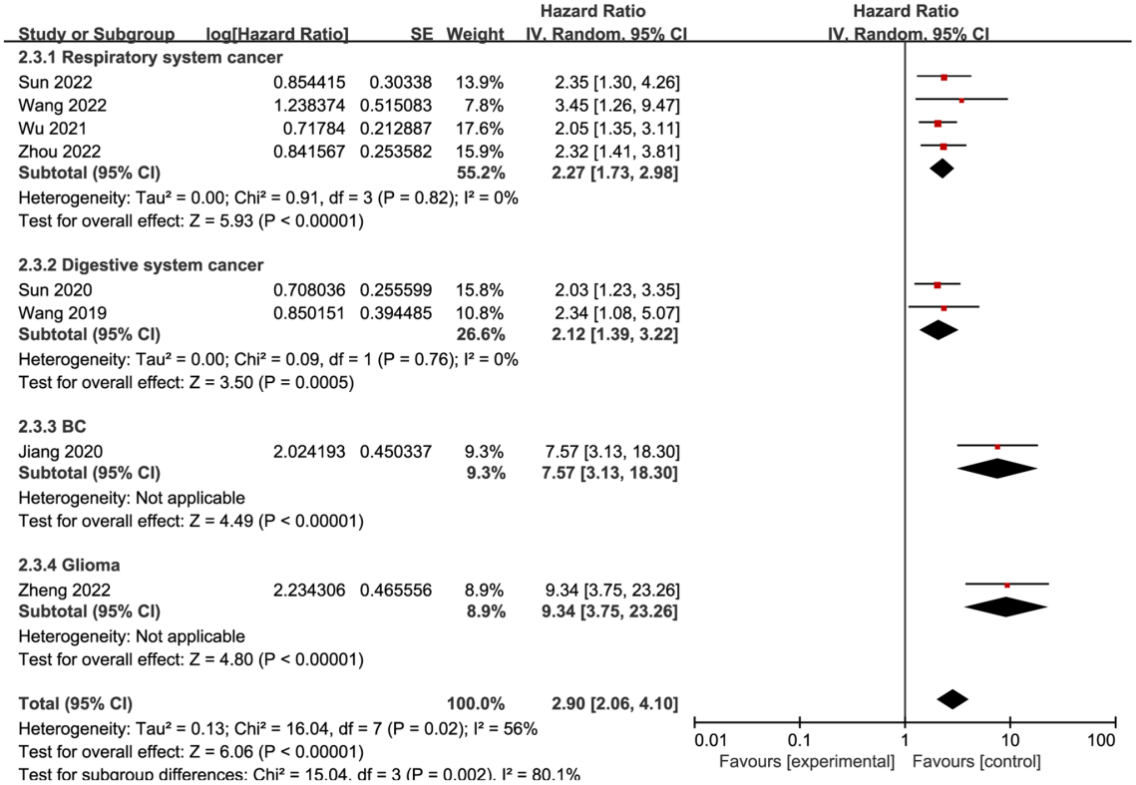
Forest plot of the pooled OS for subgroup analysis.

Eight studies reported that NCAPG upregulation was associated with tumors. Therefore, a pooled analysis of the eight studies was performed. As shown in Figure 2, a random model was used because of the absence of obvious heterogeneity (I^2^ = 56%, *P* = 0.02). The pooled results suggested that high NCAPG expression rates were correlated with worse OS in patients with different cancers (HR = 2.90, 95% confidence interval (CI) = 2.06–4.10, *P* < 0.00001).

Due to the existence of heterogeneity, a subgroup analysis was performed to further explore the impact of high NCAPG expression on survival in different types of cancer (Figure 2). Four studies [5, 9, 25, 26] investigated cancers of the respiratory system, two studies [8, 24] investigated cancers of the digestive system, and the remaining two studies investigated other types of cancers, namely, glioma [12] and breast cancer [6]. According to the forest plot, upregulation of NCAPG expression in cancer tissues was related to worse OS regardless of the group (respiratory cancer, HR = 2.27, 95% CI = 1.73–2.98, *P* < 0.00001; digestive cancer, HR = 2.12, 95% CI = 1.39–3.22, *P* = 0.0005; breast cancer, HR = 7.57, 95% CI = 3.13–18.30, *P* < 0.00001; glioma, HR = 9.34, 95% CI = 3.75–23.26, *P* < 0.00001).

### Correlation of NCAPG expression with clinicopathological parameters

**Figure 3:**
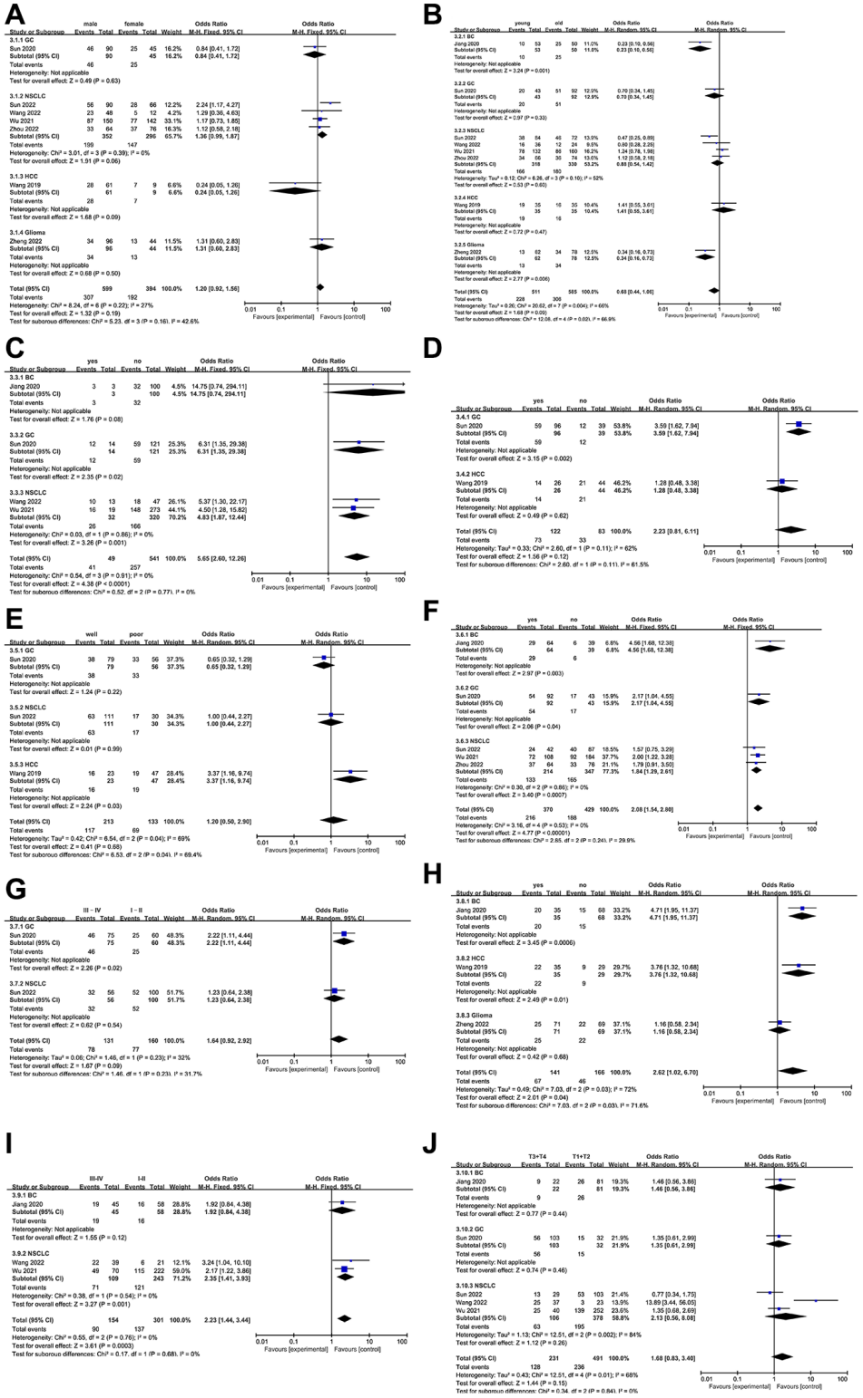
**Forest plot of the relationship between high NCAPG expression and clinicopathological parameters.** (**A**) gender, (**B**) age, (**C**) distant metastasis, (**D**) vascular invasion, (**E**) differentiation, (**F**) lymph node metastasis, (**G**) TNM stage, (**H**) relapse, (**I**) clinical stage, (**J**) T classification.

As all studies included in this meta-analysis reported clinicopathological parameters, we analyzed the high expression of NCAPG and these parameters. As shown in Figure 3, the upregulation of NCAPG was not significantly correlated with gender (male vs. female, odds ratio (OR) = 1.20, 95% CI = 0.92–1.56, *P* = 0.19, fixed model; Figure 3A), age (young vs. old, OR = 0.68, 95% CI = 0.44–1.06, *P* = 0.09, random model; Figure 3B), vascular invasion (yes vs. no, OR = 2.23, 95% CI = 0.81–6.11, *P* = 0.12, random model; Figure 3D), differentiation (well differentiated vs. poorly differentiated, OR = 1.20, 95% CI = 0.50–2.90, *P* = 0.68, fixed model; Figure 3E), TNM stage (III–IV vs. I–II, OR = 1.64, 95% CI = 0.92–2.92, *P* = 0.09, random model; Figure 3G), or T classification (T3+T4 vs. T1+T2, OR = 1.68, 95% CI = 0.83–3.40, *P* = 0.15, random model; Figure 3J), although it was correlated with distant metastasis (yes vs. no, OR = 5.65, 95% CI 2.60–12.26, *P* < 0.0001, fixed model; Figure 3C), lymph node metastasis (yes vs. no, OR = 2.08, 95% CI = 1.54–2.80, *P* < 0.00001, fixed model; Figure 3F), relapse (yes vs. no, OR = 2.62, 95% CI = 1.02–6.70, *P* = 0.04, random model; Figure 3H), and clinical stage (III–IV vs. I–II, OR = 2.23, 95% CI = 1.44–3.44, *P* = 0.0003, fixed model; Figure 3I). The results of database validation of the clinicopathological characteristics and NCAPG expression data (Supplementary Figure 1) are consistent with most of our previous meta-analysis results.

### Sensitivity analysis and publication bias

**Figure 4:**
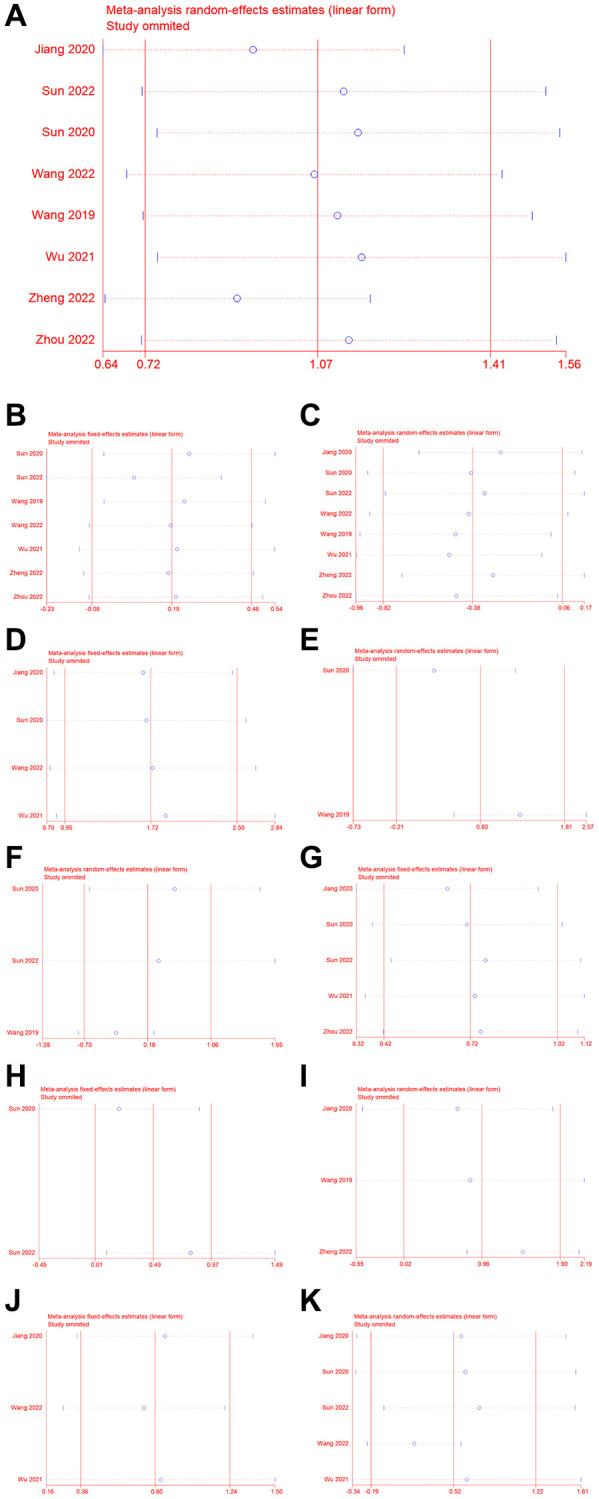
**Sensitivity analysis.** (**A**) OS, (**B**) gender, (**C**) age, (**D**) distant metastasis, (**E**) vascular invasion, (**F**) differentiation, (**G**) lymph node metastasis, (**H**) TNM stage, (**I**) relapse, (**J**) clinical stage, (**K**) T classification.

To verify the robustness of the results, we performed a sensitivity analysis (Figure 4). Removal of one or more articles did not significantly affect the results, indicating that the results were relatively stable. Begg’s and Egger’s tests indicated publication bias for OS and distant metastasis (Table 2, Supplementary Figure 2). We analyzed the stability of the study further using the trim-and-fill method. For OS, the results indicated that the estimated number of missing studies was 0 and the adjusted HR was 2.90 (95% CI = 2.06–4.10, *P* < 0.001), which indicated that upregulation of NCAPG was associated with poorer OS, suggesting that our result is reliable. In addition, for distant metastases, two studies were estimated to be missing and the adjusted OR was 4.85 (95% CI = 2.48–9.58, *P* < 0.001), which indicated a reliable result.

**Table 2:** Results of Begg’s and Egger’s tests for publication bias.

**Analysis type**	**Begg’s test**		**Egger’s test**	
	**Z**	***P***	***t***	***P***
OS	2.60	**0.009**	3.13	**0.020**
Gender (male vs. female)	0.90	0.368	−1.08	0.328
Age (young vs. old)	0.62	0.536	−1.16	0.291
Distant metastasis (yes vs. no)	1.70	0.089	6.99	**0.020**
Vascular invasion (yes vs. no)	0.00	1.000	NA	NA
Differentiation (well vs. poor)	1.04	0.296	12.42	0.051
Lymph node metastasis (yes vs. no)	0.24	0.806	1.15	0.332
TNM stage (III−IV vs. I−II)	0.00	1.000	NA	NA
Relapse (yes vs. no)	0.00	1.000	1.59	0.357
Clinical stage (III−IV vs. I−II)	0.00	1.000	0.79	0.573
T classification (T3+T4 vs. T1+T2)	1.22	0.221	2.27	0.108

If *P* < 0.05, the results are in bold. Abbreviation: NA: Not available.

### Validation of NCAPG expression against public databases

**Figure 5:**
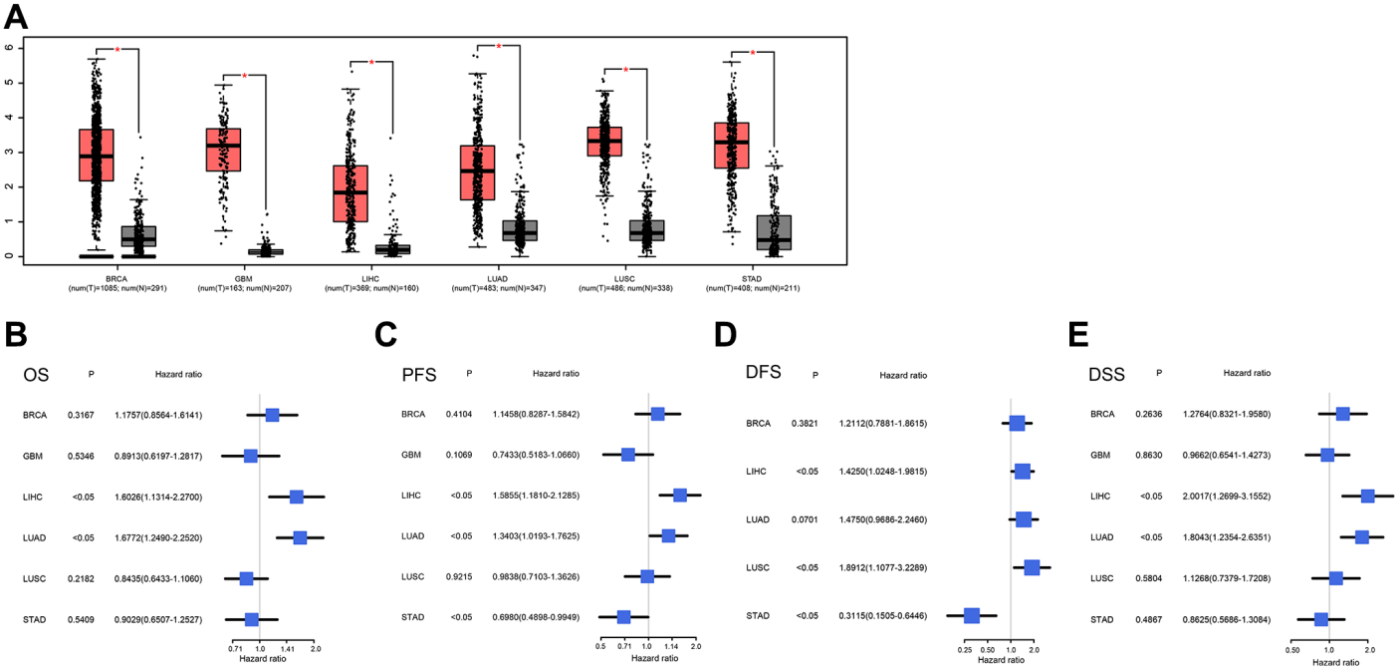
(**A**) Expression levels of NCAPG in cancer tissues and normal tissues in GEPIA2. From left to right are gastric cancer (STAD), lung cancer (LUAD), liver cancer (LIHC), glioma (GBM) and breast cancer (BRCA). The red box represents the expression level of NCAPG in cancer tissues; the gray box represents the expression level of NCAPG in normal tissues, the screening criteria were log2FC|>1 and *P* < 0.01, (**B**) OS of BRCA, GBM, LIHC, LUAD, LUSC and STAD, (**C**) PFS of BRCA, GBM, LIHC, LUAD, LUSC and STAD, (**D**) DFS of BRCA, LIHC, LUAD, LUSC and STAD, (**E**) BRCA, GBM, LIHC, LUAD, LUSC and STAD's DSS.

**Figure 6:**
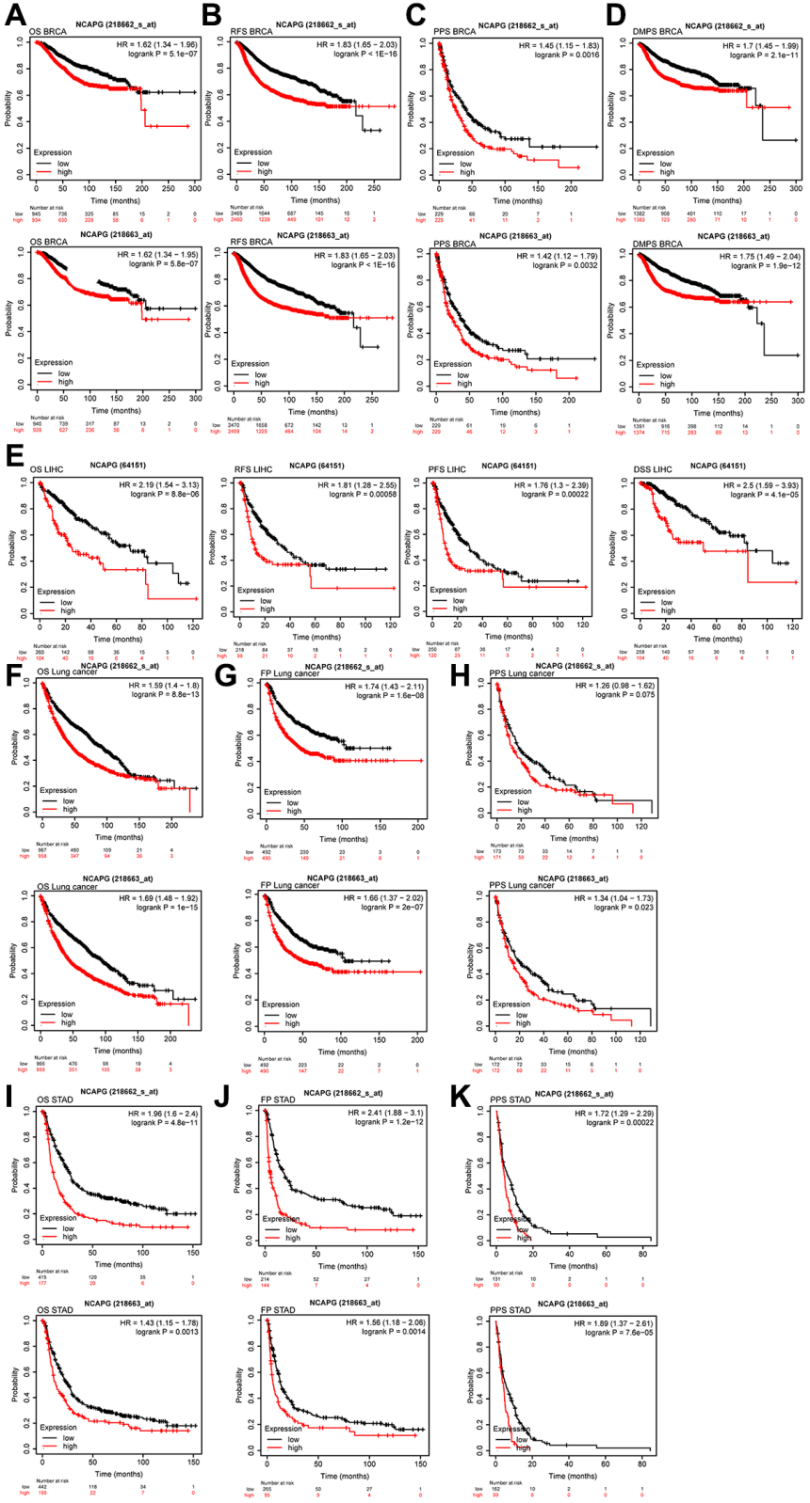
(**A**) OS of NCAPG (218663-s-at) in BRCA (*n* = 1879), OS of NCAPG (218663-at) in BRCA (*n* = 1879), (**B**) RFS of NCAPG (218663-s-at) in BRCA (*n* = 4929), RFS of NCAPG (218663-at) in BRCA (*n* = 4929), (**C**) PPS of NCAPG (218663-s-at) in BRCA (*n* = 458), PPS of NCAPG (218663-at) in BRCA (*n* = 458)(**D**) DMPS of NCAPG (218663-s-at) in BRCA (*n* = 2765), DMPS of NCAPG (218663-at) in BRCA (*n* = 2765), (**E**) OS (*n* = 364), PFS (*n* = 316), PFS (*n* = 370)and DSS (*n* = 362)of NCAPG in LIHC, (**F**) OS of NCAPG (218663-s-at) in lung cancer (*n* = 1925), OS of NCAPG (218663-at) in lung cancer (*n* = 1925), (**G**) FP of NCAPG (218663-s-at) in lung cancer (*n* = 982), FP of NCAPG (218663-at) in lung cancer (*n* = 982), (**H**) PPS of NCAPG (218663-s-at) in lung cancer (*n* = 344), PPS of NCAPG (218663-at) in lung cancer (*n* = 344), (**I**) OS of NCAPG (218663-s-at) in STAD (*n* = 592), OS of NCAPG (218663-at) in STAD (*n* = 592), (**J**) FP of NCAPG (218663-s-at) in STAD (*n* = 358), FP of NCAPG (218663-at) in STAD (*n* = 358), (**K**) PPS of NCAPG (218663-s-at) in STAD (*n* = 221), PPS of NCAPG (218663-at) in STAD (*n* = 221).

We evaluated the NCAPG expression levels and performed a survival analysis in various cancers using the GEPIA2.0 database to validate our results. The findings showed that, compared with normal tissues, the expression of NCAPG was significantly upregulated in tumors, including BRCA, GBM, LIHC, LUAD, LUSC, and STAD (Figure 5A). We next performed a univariate Cox survival analysis on the above tumors, and it showed that the OS of LIHC and LUAD was related to NCAPG (Figure 5B). The progression-free survival (PFS) of BRCA, LIHC, LUAD, and STAD was related to NCAPG (Figure 5C). The relapse-free survival (RFS) of LIHC, LUSC, and STAD was related to NCAPG (Figure 5D), and the disease-specific survival (DSS) of LIHC and LUAD was related to NCAPG (Figure 5E). We then performed receiver operating characteristic (ROC) survival analysis of the data on the above tumors (Supplementary Figure 3), and the area under the curve of GBM, LIHC, and STAD was greater than 0.7. Therefore, we concluded that NCAPG may be a good prognostic indicator of various cancers. In addition, we analyzed the survival prognosis of the above tumors using the Kaplan-Meier plotter database based on the median cutoff value of NCAPG expression (including probes 218663_at and 218663_s_at) in cancer. The results showed that NCAPG expression can be used to predict OS in breast cancer (*P* < 0.01; Figure 6A), RFS (*P* < 0.01; Figure 6B), PPS (*P* < 0.01; Figure 6C), DMFS (*P* < 0.01; Figure 6D), liver cancer, PFS, PFS, and DSS (*P* < 0.01; Figure 6E), lung cancer (*P* < 0.01; Figure 6F), FP (*P* < 0.01; Figure 6G), and PPS (*P* < 0.01; Figure 6H), gastric cancer (*P* < 0.01; Figure 6I), FP (*P* < 0.01; Figure 6J,), and PPS (*P* < 0.01; Figure 6K). Moreover, the findings were validated against multiple cancer datasets using the PrognoScan database. We collected 29 datasets on breast and lung cancers. As shown in Table 3, NCAPG expression in these tumors significantly affected prognosis-related indicators, such as OS, DSS, RFS, and PFS (*P* < 0.05).

**Table 3:** NCAPG-related cancer GEO database classifications.

**Dataset**	**Cancer type**	**Prognostic factor**	**Cox *P*-value**	**ln (HR)**	**HR (95% CI)**
GSE5287	Bladder cancer	Overall Survival	0.043472	0.555305	1.74 (1.02–2.99)
GSE5287	Bladder cancer	Overall Survival	0.328603	0.223676	1.25 (0.80–1.96)
GSE13507	Bladder cancer	Overall Survival	0.000399636	0.380311	1.46 (1.19–1.81)
GSE13507	Bladder cancer	Disease Specific Survival	7.89E-05	0.698904	2.01 (1.42–2.85)
GSE12417-GPL96	Blood cancer	Overall Survival	0.294458	−0.153815	0.86 (0.64–1.14)
GSE12417-GPL96	Blood cancer	Overall Survival	0.391317	−0.121301	0.89 (0.67–1.17)
GSE12417-GPL570	Blood cancer	Overall Survival	0.924132	−0.0155483	0.98 (0.71–1.36)
GSE12417-GPL570	Blood cancer	Overall Survival	0.895552	0.0205993	1.02 (0.75–1.39)
GSE5122	Blood cancer	Overall Survival	0.852157	−0.025255	0.98 (0.75–1.27)
GSE5122	Blood cancer	Overall Survival	0.980633	−0.00489207	1.00 (0.67–1.48)
GSE8970	Blood cancer	Overall Survival	0.116843	−0.317877	0.73 (0.49–1.08)
GSE8970	Blood cancer	Overall Survival	0.14972	−0.274213	0.76 (0.52–1.10)
GSE4475	Blood cancer	Overall Survival	0.0095096	−0.464977	0.63 (0.44–0.89)
E-TABM-346	Blood cancer	Overall Survival	0.865305	−0.0630235	0.94 (0.45–1.94)
E-TABM-346	Blood cancer	Overall Survival	0.951871	0.0174584	1.02 (0.58–1.79)
E-TABM-346	Blood cancer	Event Free Survival	0.707167	−0.126166	0.88 (0.46–1.70)
E-TABM-346	Blood cancer	Event Free Survival	0.998653	0.000438628	1.00 (0.60–1.66)
GSE16131-GPL96	Blood cancer	Overall Survival	0.86364	0.0296222	1.03 (0.73–1.44)
GSE16131-GPL96	Blood cancer	Overall Survival	0.774936	−0.0475906	0.95 (0.69–1.32)
GSE2658	Blood cancer	Disease Specific Survival	0.0835894	0.410555	1.51 (0.95–2.40)
GSE2658	Blood cancer	Disease Specific Survival	0.00039476	0.382343	1.47 (1.19–1.81)
GSE4271	Brain cancer	Overall Survival	0.00174277	0.58775	1.80 (1.25–2.60)
GSE7696	Brain cancer	Overall Survival	0.993869	0.00119241	1.00 (0.74–1.36)
GSE7696	Brain cancer	Overall Survival	0.567686	0.0919879	1.10 (0.80–1.50)
GSE16581	Brain cancer	Overall Survival	0.844517	0.141127	1.15 (0.28–4.72)
GSE16581	Brain cancer	Overall Survival	0.542849	0.658295	1.93 (0.23–16.10)
GSE19615	Breast cancer	Distant Metastasis Free Survival	0.375869	0.346581	1.41 (0.66–3.05)
GSE19615	Breast cancer	Distant Metastasis Free Survival	0.53393	0.279045	1.32 (0.55–3.18)
GSE12276	Breast cancer	Relapse Free Survival	0.000105043	0.427798	1.53 (1.24–1.90)
GSE6532-GPL570	Breast cancer	Distant Metastasis Free Survival	0.115457	0.314844	1.37 (0.93–2.03)
GSE6532	Breast cancer	Distant Metastasis Free Survival	0.0361386	0.384826	1.47 (1.03–2.11)
GSE6532-GPL570	Breast cancer	Relapse Free Survival	0.115457	0.314844	1.37 (0.93–2.03)
GSE6532	Breast cancer	Relapse Free Survival	0.0361386	0.384826	1.47 (1.03–2.11)
GSE9195	Breast cancer	Distant Metastasis Free Survival	0.434869	0.261618	1.30 (0.67–2.50)
GSE9195	Breast cancer	Distant Metastasis Free Survival	0.165806	0.510274	1.67 (0.81–3.43)
GSE9195	Breast cancer	Relapse Free Survival	0.224745	0.397302	1.49 (0.78–2.83)
GSE9195	Breast cancer	Relapse Free Survival	0.499771	0.202634	1.22 (0.68–2.21)
GSE12093	Breast cancer	Distant Metastasis Free Survival	0.0122697	0.923643	2.52 (1.22–5.19)
GSE11121	Breast cancer	Distant Metastasis Free Survival	0.00994604	0.574941	1.78 (1.15–2.75)
GSE1378	Breast cancer	Relapse Free Survival	0.755055	0.0639828	1.07 (0.71–1.59)
GSE1379	Breast cancer	Relapse Free Survival	0.728436	0.0804905	1.08 (0.69–1.71)
GSE2034	Breast cancer	Distant Metastasis Free Survival	0.00249838	0.515644	1.67 (1.20–2.34)
GSE1456	Breast cancer	Overall Survival	0.000786964	1.06803	2.91 (1.56–5.43)
GSE1456	Breast cancer	Disease Specific Survival	0.000648569	0.983901	2.67 (1.52–4.71)
GSE1456	Breast cancer	Relapse Free Survival	0.000279683	1.15084	3.16 (1.70–5.88)
GSE7378	Breast cancer	Disease Free Survival	0.0335011	0.654287	1.92 (1.05–3.52)
GSE7378	Breast cancer	Disease Free Survival	0.0709317	0.554641	1.74 (0.95–3.18)
E-TABM-158	Breast cancer	Distant Metastasis Free Survival	0.681173	0.0873489	1.09 (0.72–1.66)
E-TABM-158	Breast cancer	Overall Survival	0.203745	−0.227607	0.80 (0.56–1.13)
E-TABM-158	Breast cancer	Relapse Free Survival	0.361575	−0.17199	0.84 (0.58–1.22)
E-TABM-158	Breast cancer	Disease Specific Survival	0.0899647	−0.399162	0.67 (0.42–1.06)
E-TABM-158	Breast cancer	Overall Survival	0.361575	−0.17199	0.84 (0.58–1.22)
E-TABM-158	Breast cancer	Distant Metastasis Free Survival	0.757249	0.0666703	1.07 (0.70–1.63)
E-TABM-158	Breast cancer	Relapse Free Survival	0.203745	−0.227607	0.80 (0.56–1.13)
E-TABM-158	Breast cancer	Disease Specific Survival	0.0351901	−0.454509	0.63 (0.42–0.97)
GSE3494	Breast cancer	Disease Specific Survival	0.00163843	0.590531	1.80 (1.25–2.61)
GSE4922	Breast cancer	Disease Free Survival	4.93E-05	0.808544	2.24 (1.52–3.32)
GSE2990	Breast cancer	Distant Metastasis Free Survival	0.0759889	0.351849	1.42 (0.96–2.10)
GSE2990	Breast cancer	Relapse Free Survival	0.105799	0.500621	1.65 (0.90–3.03)
GSE2990	Breast cancer	Relapse Free Survival	0.123393	0.239449	1.27 (0.94–1.72)
GSE2990	Breast cancer	Distant Metastasis Free Survival	0.00239721	0.895966	2.45 (1.37–4.37)
GSE2990	Breast cancer	Distant Metastasis Free Survival	0.0796503	0.412016	1.51 (0.95–2.39)
GSE2990	Breast cancer	Relapse Free Survival	0.1058	0.313035	1.37 (0.94–2.00)
GSE2990	Breast cancer	Distant Metastasis Free Survival	0.142013	0.556377	1.74 (0.83–3.67)
GSE2990	Breast cancer	Relapse Free Survival	0.00369961	0.72696	2.07 (1.27–3.38)
GSE7390	Breast cancer	Distant Metastasis Free Survival	0.17505	0.143945	1.15 (0.94–1.42)
GSE7390	Breast cancer	Overall Survival	0.0654623	0.211501	1.24 (0.99–1.55)
GSE7390	Breast cancer	Relapse Free Survival	0.217378	0.140286	1.15 (0.92–1.44)
GSE7390	Breast cancer	Distant Metastasis Free Survival	0.279784	0.152457	1.16 (0.88–1.54)
GSE7390	Breast cancer	Relapse Free Survival	0.133969	0.127109	1.14 (0.96–1.34)
GSE7390	Breast cancer	Overall Survival	0.203224	0.191366	1.21 (0.90–1.63)
GSE12945	Colorectal cancer	Disease Free Survival	0.390324	0.626282	1.87 (0.45–7.81)
GSE12945	Colorectal cancer	Overall Survival	0.115402	0.789413	2.20 (0.82–5.88)
GSE12945	Colorectal cancer	Disease Free Survival	0.672657	−0.773745	0.46 (0.01–16.70)
GSE12945	Colorectal cancer	Overall Survival	0.16887	1.33458	3.80 (0.57–25.43)
GSE17536	Colorectal cancer	Disease Specific Survival	0.589054	−0.130516	0.88 (0.55–1.41)
GSE17536	Colorectal cancer	Disease Specific Survival	0.828392	0.0428782	1.04 (0.71–1.54)
GSE17536	Colorectal cancer	Overall Survival	0.856783	0.0390747	1.04 (0.68–1.59)
GSE17536	Colorectal cancer	Overall Survival	0.262151	0.198586	1.22 (0.86–1.73)
GSE17536	Colorectal cancer	Disease Free Survival	0.109121	−0.506703	0.60 (0.32–1.12)
GSE17536	Colorectal cancer	Disease Free Survival	0.268629	−0.263513	0.77 (0.48–1.23)
GSE14333	Colorectal cancer	Disease Free Survival	0.0827771	−0.427442	0.65 (0.40–1.06)
GSE14333	Colorectal cancer	Disease Free Survival	0.021572	−0.444143	0.64 (0.44–0.94)
GSE17537	Colorectal cancer	Overall Survival	0.908953	0.0357876	1.04 (0.56–1.91)
GSE17537	Colorectal cancer	Overall Survival	0.616503	0.136126	1.15 (0.67–1.95)
GSE17537	Colorectal cancer	Disease Free Survival	0.713261	0.125822	1.13 (0.58–2.22)
GSE17537	Colorectal cancer	Disease Free Survival	0.33555	0.294475	1.34 (0.74–2.44)
GSE17537	Colorectal cancer	Disease Specific Survival	0.142937	0.741175	2.10 (0.78–5.66)
GSE17537	Colorectal cancer	Disease Specific Survival	0.0989951	0.789785	2.20 (0.86–5.63)
GSE22138	Eye cancer	Distant Metastasis Free Survival	0.440403	0.174746	1.19 (0.76–1.86)
GSE22138	Eye cancer	Distant Metastasis Free Survival	0.0942372	0.746158	2.11 (0.88–5.05)
GSE2837	Head and neck cancer	Relapse Free Survival	0.160158	−0.644551	0.52 (0.21–1.29)
jacob-00182-CANDF	Lung cancer	Overall Survival	0.426986	0.197876	1.22 (0.75–1.99)
jacob-00182-CANDF	Lung cancer	Overall Survival	0.184801	0.237298	1.27 (0.89–1.80)
jacob-00182-HLM	Lung cancer	Overall Survival	0.565996	0.0919948	1.10 (0.80–1.50)
jacob-00182-HLM	Lung cancer	Overall Survival	0.572148	0.0813385	1.08 (0.82–1.44)
jacob-00182-MSK	Lung cancer	Overall Survival	0.0865412	0.267983	1.31 (0.96–1.78)
jacob-00182-MSK	Lung cancer	Overall Survival	0.0414568	0.367399	1.44 (1.01–2.06)
GSE13213	Lung cancer	Overall Survival	0.00524186	0.3675	1.44 (1.12–1.87)
GSE31210	Lung cancer	Relapse Free Survival	3.00E-05	0.623234	1.86 (1.39–2.50)
GSE31210	Lung cancer	Overall Survival	0.00404387	0.597241	1.82 (1.21–2.73)
jacob-00182-UM	Lung cancer	Overall Survival	0.158649	0.165838	1.18 (0.94–1.49)
jacob-00182-UM	Lung cancer	Overall Survival	0.313668	0.0915618	1.10 (0.92–1.31)
GSE3141	Lung cancer	Overall Survival	0.251246	0.222022	1.25 (0.85–1.82)
GSE3141	Lung cancer	Overall Survival	0.439787	0.186023	1.20 (0.75–1.93)
GSE14814	Lung cancer	Overall Survival	0.347644	0.275749	1.32 (0.74–2.34)
GSE14814	Lung cancer	Disease Specific Survival	0.213061	0.40874	1.50 (0.79–2.86)
GSE14814	Lung cancer	Disease Specific Survival	0.163201	0.63031	1.88 (0.77–4.56)
GSE14814	Lung cancer	Overall Survival	0.339429	0.394737	1.48 (0.66–3.34)
GSE8894	Lung cancer	Relapse Free Survival	0.194985	0.138056	1.15 (0.93–1.41)
GSE8894	Lung cancer	Relapse Free Survival	0.0887164	0.16656	1.18 (0.98–1.43)
GSE4573	Lung cancer	Overall Survival	0.102369	0.458103	1.58 (0.91–2.74)
GSE4573	Lung cancer	Overall Survival	0.238414	0.356487	1.43 (0.79–2.58)
GSE17710	Lung cancer	Relapse Free Survival	0.257306	0.342858	1.41 (0.78–2.55)
GSE17710	Lung cancer	Relapse Free Survival	0.203291	0.389413	1.48 (0.81–2.69)
GSE17710	Lung cancer	Overall Survival	0.168822	0.435278	1.55 (0.83–2.87)
GSE17710	Lung cancer	Overall Survival	0.127973	0.489001	1.63 (0.87–3.06)
GSE9891	Ovarian cancer	Overall Survival	0.0458145	0.171656	1.19 (1.00–1.41)
DUKE-OC	Ovarian cancer	Overall Survival	0.190803	−0.132614	0.88 (0.72–1.07)
DUKE-OC	Ovarian cancer	Overall Survival	0.774339	−0.0393488	0.96 (0.73–1.26)
GSE26712	Ovarian cancer	Overall Survival	0.104731	−0.203939	0.82 (0.64–1.04)
GSE26712	Ovarian cancer	Overall Survival	0.493253	−0.135951	0.87 (0.59–1.29)
GSE26712	Ovarian cancer	Disease Free Survival	0.817429	−0.0412868	0.96 (0.68–1.36)
GSE26712	Ovarian cancer	Disease Free Survival	0.112295	−0.181627	0.83 (0.67–1.04)
GSE17260	Ovarian cancer	Overall Survival	0.374023	0.125722	1.13 (0.86–1.50)
GSE17260	Ovarian cancer	Progression Free Survival	0.249324	0.123087	1.13 (0.92–1.39)
GSE14764	Ovarian cancer	Overall Survival	0.20335	0.297358	1.35 (0.85–2.13)
GSE14764	Ovarian cancer	Overall Survival	0.330025	0.268734	1.31 (0.76–2.25)
GSE19234	Skin cancer	Overall Survival	0.00485285	1.28566	3.62 (1.48–8.85)
GSE30929	Soft tissue cancer	Distant Recurrence Free Survival	0.000134506	0.466765	1.59 (1.26–2.03)

### Molecular role and functional enrichment analysis results of NCAPG

**Figure 7:**
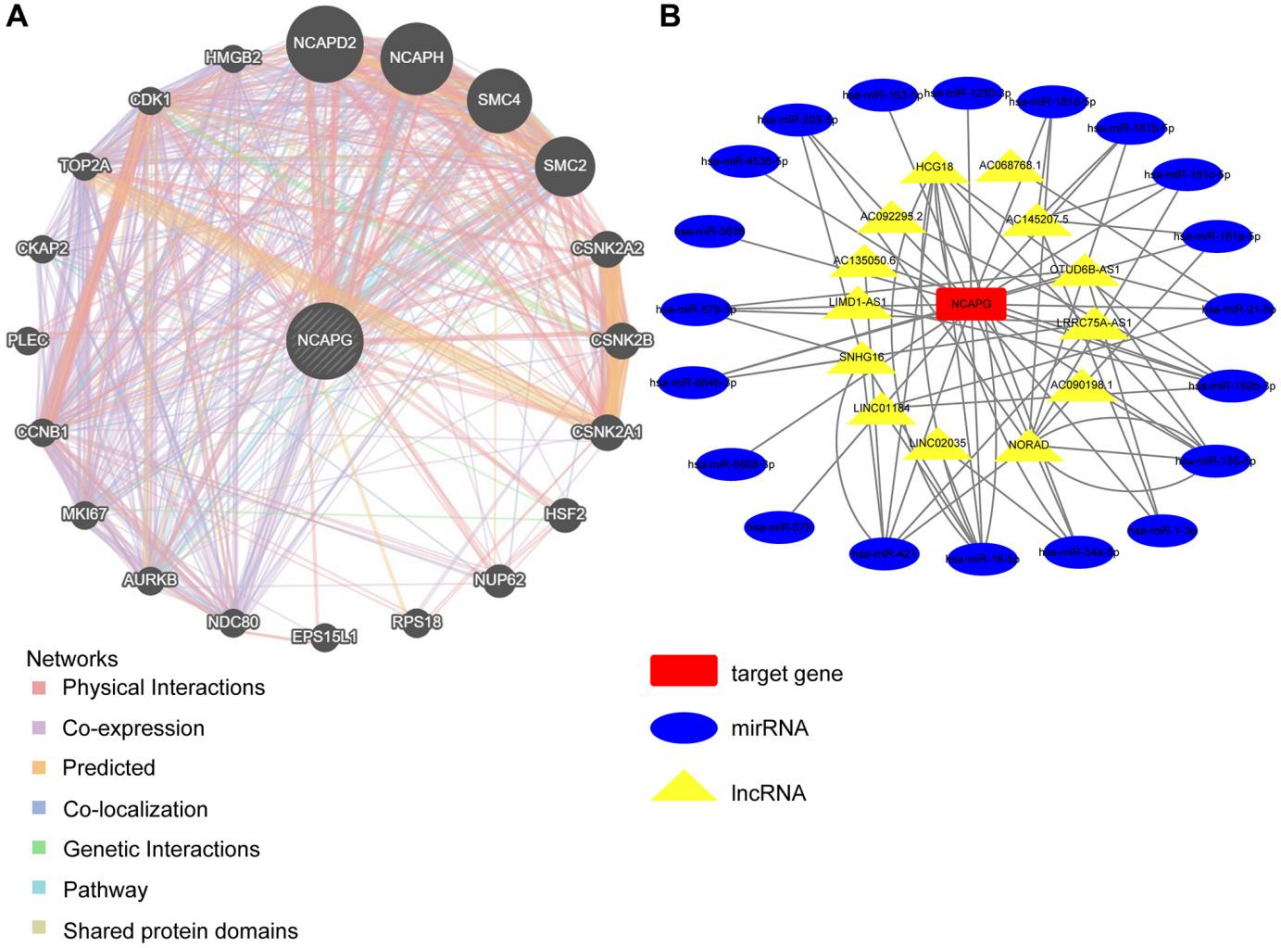
Network analysis between NCAPG and target genes (**A**) PPI network for KIF23 was constructed in Gene MANIA, Different colors of the network edge indicate the bioinformatics methods applied: physical interaction, co-expression, predicted, co-localization, pathway, genetic interaction and shared protein domains. Abbreviation: PPI: protein–protein interaction. (**B**) The relationship between NCAPG and non-coding RNA, the red square represents the target gene NCAPG, the blue oval represents miRNA, and the yellow triangle represents lncRNA.

**Figure 8:**
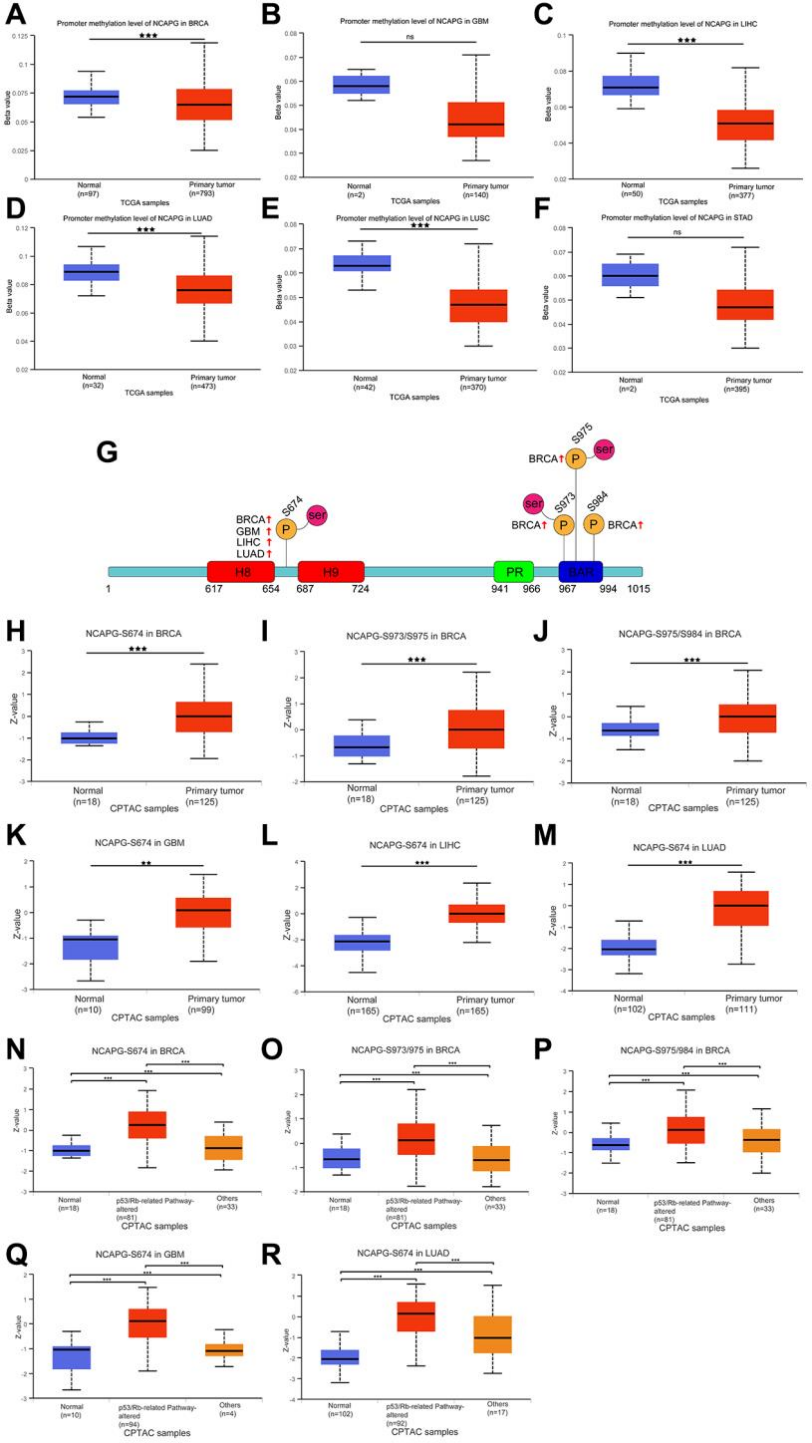
DNA methylation features of NCAPG in BRCA (**A**), GBM (**B**), LIHC (**C**), LUAD (**D**), LUSC (**E**) and STAD (**F**). Phosphorylation of NCAPG in several selected cancers according to the CPTAC database. (**G**) The schematic diagram and phosphorylation sites of the NCAPG protein are shown. The phosphorylation of NCAPG at S674, S973, S975 and 984 in BRCA (**H**–**J**), S674 in GBM (**K**), S674 in LIHC (**L**), S674 in LUAD (**M**). The P53 pathway phosphorylation of NCAPG at S674, S973, S975 and 984 in BRCA (**N**–**P**), S674 in GBM (**Q**), S674 in LUAD (**R**), from the UALCAN database. ^*^p < 0:05, ^**^p < 0:01, and ^***^p < 0:001, Abbreviation: ns: No statistical significance.

We used the GeneMANIA database for the protein-molecular interaction analysis of NCAPG and its related molecules, such as NCAPG2, NCAPH, and SMC4 (Figure 7A). Gene Ontology (GO) and Kyoto Encyclopedia of Genes and Genomes (KEGG) functional and pathway enrichment analyses of NCAPG were performed using the STRING database. The most abundant GO terms were nuclear division, cell division, and cell cycle process (Table 4). In addition, the KEGG pathway analysis confirmed that these co-expressed genes were significantly involved in the p53 signaling pathway, cell cycle, and cellular senescence (Table 4). These results indicate that NCAPG is involved in the biological pathways of cancer. Additionally, we used the multiMiR package to identify NCAPG-related miRNAs and lncRNAs (the screening criterion for miRNAs was a predicted cutoff of 500,000, and the screening criteria for lncRNAs were lnc_mi$pancancerNum>10 and lnc_mi$clipExpNum>4) that may interact with NCAPG (Figure 7B). We identified 20 miRNAs and 13 lncRNAs, which could provide direction for future experimental designs. DNA methylation directly affects the occurrence and progression of cancers. We used the UALCAN database to investigate the DNA methylation of NCAPG. Our results showed that NCAPG methylation levels were significantly reduced in BRCA, GBM, LIHC, LUAD, and LUSC tissues compared to normal tissues (Figure 8A–8F), which may explain the difference in NCAPG expression between BRCA, GBM, LIHC, LUAD, and LUSC tissues and normal tissues. Post-translational modification is a key molecular mechanism underlying NCAPG activation. Therefore, we examined the changes in NCAPG phosphorylation levels between tumor tissues and normal tissues. The Clinical Proteomic Tumor Analysis Consortium database includes four cancers: BRCA, GBM, LIHC, and LUAD. Compared with the normal samples, the phosphorylation levels at 674/973/975/984 of NCAPG were higher in BRCA, GBM, LIHC, and LUAD, respectively (Figure 8G). The specific results are shown in Figure 8H–8M. Since the p53 signaling pathway is highly enriched, we also explored the phosphorylation of NCAPG in this pathway, and the results are shown in Figure 8N–8R.

**Table 4:** Functional enrichment analysis and pathway enrichment analysis of NCAPG genes.

**GO ID**	**Term description**	**Ontology**	**Count**	**False discovery rate**
GO:0000280	Nuclear division	BP	11	1.26E-16
GO:0140014	Mitotic nuclear division	BP	10	1.26E-16
GO:0030261	Chromosome condensation	BP	8	5.29E-16
GO:0051301	Cell division	BP	11	7.60E-15
GO:0000070	Mitotic sister chromatid segregation	BP	8	4.14E-13
GO:0007076	Mitotic chromosome condensation	BP	6	8.07E-13
GO:0022402	Cell cycle process	BP	11	7.24E-12
GO:0010032	Meiotic chromosome condensation	BP	5	1.78E-11
GO:1903046	Meiotic cell cycle process	BP	6	1.29E-07
GO:0051276	Chromosome organization	BP	9	1.48E-07
GO:0007077	Mitotic nuclear envelope disassembly	BP	3	3.34E-05
GO:0022414	Reproductive process	BP	8	5.10E-05
GO:0051304	Chromosome separation	BP	3	0.00033
GO:0140013	Meiotic nuclear division	BP	4	0.00049
GO:1905448	Positive regulation of mitochondrial atp synthesis coupled electron transport	BP	2	0.0023
GO:0051987	Positive regulation of attachment of spindle microtubules to kinetochore	BP	2	0.0029
GO:0031145	Anaphase-promoting complex-dependent catabolic process	BP	3	0.0046
GO:0045132	Meiotic chromosome segregation	BP	3	0.0051
GO:0035404	Histone-serine phosphorylation	BP	2	0.0082
GO:0055015	Ventricular cardiac muscle cell development	BP	2	0.0082
GO:0034501	Protein localization to kinetochore	BP	2	0.01
GO:0051782	Negative regulation of cell division	BP	2	0.0139
GO:0000086	G2/M transition of mitotic cell cycle	BP	3	0.0145
GO:0007292	Female gamete generation	BP	3	0.0145
GO:0051383	Kinetochore organization	BP	2	0.0145
GO:0007051	Spindle organization	BP	3	0.0162
GO:0007093	Mitotic cell cycle checkpoint	BP	3	0.0197
GO:0045931	Positive regulation of mitotic cell cycle	BP	3	0.0197
GO:0060045	Positive regulation of cardiac muscle cell proliferation	BP	2	0.0247
GO:0000226	Microtubule cytoskeleton organization	BP	4	0.0261
GO:0010971	Positive regulation of g2/m transition of mitotic cell cycle	BP	2	0.0261
GO:0018105	Peptidyl-serine phosphorylation	BP	3	0.0261
GO:0010389	Regulation of g2/m transition of mitotic cell cycle	BP	3	0.0338
GO:1901991	Negative regulation of mitotic cell cycle phase transition	BP	3	0.0412
GO:0000796	Condensin complex	CC	6	3.01E-14
GO:0000793	Condensed chromosome	CC	8	3.53E-11
GO:0000799	Nuclear condensin complex	CC	4	2.14E-09
GO:0000794	Condensed nuclear chromosome	CC	6	4.97E-09
GO:0000797	Condensin core heterodimer	CC	3	7.47E-07
GO:0000779	Condensed chromosome, centromeric region	CC	5	1.06E-06
GO:0098687	Chromosomal region	CC	6	1.80E-06
GO:0005694	Chromosome	CC	9	2.71E-06
GO:0000228	Nuclear chromosome	CC	8	6.95E-06
GO:0043232	Intracellular non-membrane-bounded organelle	CC	11	3.32E-05
GO:0000307	Cyclin-dependent protein kinase holoenzyme complex	CC	3	0.00021
GO:0097125	Cyclin b1-cdk1 complex	CC	2	0.00021
GO:0005813	Centrosome	CC	5	0.00087
GO:0032991	Protein-containing complex	CC	10	0.0011
GO:0005634	Nucleus	CC	11	0.0019
GO:0005819	Spindle	CC	4	0.0026
GO:0072686	Mitotic spindle	CC	3	0.0029
GO:0000922	Spindle pole	CC	3	0.0064
GO:0030496	Midbody	CC	3	0.0083
GO:0000780	Condensed nuclear chromosome, centromeric region	CC	2	0.0092
GO:0005829	Cytosol	CC	9	0.0132
GO:0005654	Nucleoplasm	CC	8	0.0161
GO:0005876	Spindle microtubule	CC	2	0.018
GO:0035173	Histone kinase activity	MF	3	0.00041
**KEGG ID**	**Term description**		**Count**	**False discovery rate**
hsa04115	p53 signaling pathway		3	0.0029
hsa04914	Progesterone-mediated oocyte maturation		3	0.0032
hsa04110	Cell cycle		3	0.0043
hsa04114	Oocyte meiosis		3	0.0043
hsa04218	Cellular senescence		3	0.005
hsa05170	Human immunodeficiency virus 1 infection		3	0.0101

Abbreviations: BP: Biological Process; CC: Cellular Component; ME: Molecular Function; KEGG: Kyoto Encyclopedia of Genes and Genomes.

## DISCUSSION

NCAPG expression was initially found to be correlated with the prognosis of liver cancer. Later on, the expression of NCAPG was often closely associated with the survival outcome and clinical pathology of patients with diseases such as NSCLC, renal clear cell carcinoma, breast cancer, and gastric cancer [27]. To better verify and summarize the value of this gene and avoid the errors caused by small samples or small queues, we used meta-methods and bioinformatics jointly.

First, in this study, the results of the meta-analysis showed that high expression of NCAPG is associated with poor prognosis, suggesting its role as a proto-oncogene in cancer. Second, eight studies were included in this meta-analysis. Our results suggest that cancer patients with upregulated NCAPG expression have a 2.90-fold worse OS than those with low expression. We also performed a subgroup analysis according to the different systems. The results showed that NCAPG might be a potential prognostic marker for cancers of the respiratory, digestive, and other systems. Third, from the perspective of bioinformatics, univariate cox regression analysis showed that NCAPG was a bad prognostic factor for LIHC, LUAD, and STAD. We also verified this inference using the Kaplan-Meier plotter database and found that the high expression of NCAPG was related to the poor prognosis of BRCA, LIHC, LUAD, and STAD. We calculated the relationship between the expression of NCAPG and the annual survival rate of the cancer (Supplementary Figure 3). The results showed that NCAPG could predict the survival and prognosis of GBM, LIHC, and STAD.

Additionally, we assessed the association between NCAPG expression and clinicopathological parameters. The pooled results showed that the upregulation of NCAPG was not associated with age, sex, vascular invasion, differentiation, TNM stage, and T classification but was associated with distant metastasis, lymph node metastasis, relapse, and clinical stage. The results of the sensitivity and publication bias analyses demonstrated the reliability of the results. To our knowledge, this is the first meta-analysis to demonstrate the prognostic value of NCAPG in cancer.

To further explore the relationship between NCAPG expression and the clinicopathological features of different cancers, a subgroup analysis was performed. We found that NCAPG overexpression was significantly correlated with positive lymph node metastasis in gastric cancer and NSCLC, TNM stage in hepatocellular carcinoma, age in glioma, differentiation in hepatocellular carcinoma and glioma, and vascular invasion in gastric cancer. Next, we verified the clinicopathological features of cancers with NCAPG expression using the UALCAN database (Supplementary Figure 1). The results showed that the expression of NCAPG was related to the age, lymph node metastasis, and stage of tumor patients.

How NCAPG can precisely regulate oncogenes remains unknown, but some studies have proved that it may be related to the following mechanisms. NCAPG induces epigenetic changes of tumors through a variety of signal pathways and molecules. In lung cancer, NCAPG expression activates TGF-β signaling pathway [5]. In breast cancer, it is related to the SRC/STAT3 signaling pathway [6] and p53 signaling pathway [28]. In colorectal cancer, it is associated with Wnt/β-catenin signaling pathway [29]. In hepatocellular carcinoma, it is related to activation of PI3K/AKT signaling pathway [7]. In cardiac adenocarcinoma, Wnt/β-catenin signaling pathway [30] and PI3K/AKT signaling pathway are involved [31]. In endometrial carcinoma, it is related to Wnt/β-catenin signaling pathway [32]. In oral squamous cell carcinoma, it is related to miR-378a-3p-mediated GSK-3β/β-catenin signaling [33]. In prostate cancer, it interacts with miR-99a-3p [34]. In bladder cancer, it is related to NF-κB signaling pathway [35]. In ovarian cancer, it is related to p38 MAPK signaling pathway [36].

We also analyzed the GO and KEGG pathways of NCAPG. The richest GO terms were nuclear division, cell division, and cell cycle processes, all of which are related to cancer cell proliferation. In addition, KEGG pathway analysis confirmed that NCAPG-related genes were involved in the p53 signaling pathway, cell senescence, and cell cycle, which are involved in the mechanism underlying carcinogenesis. We also explored the changes in phosphorylation and methylation of NCAPG in these tumors. Finally, we identified the miRNAs and lncRNAs related to NCAPG.

This study has a few limitations. First, a total of eight studies were included in this study, which is a relatively small sample size and may affect the accuracy of the results; therefore, additional research is needed to confirm the findings. Second, it is necessary to fully verify and clarify the role and mechanism of NCAPG in cancer through cell models *in vitro* and *in vivo*. Furthermore, some studies used the K-M curve to extract the HR of OS, which may have had an impact on the results and led to publication bias. Finally, due to the different methods used to evaluate NCAPG expression and different cutoff value standards, statistical errors may have been introduced.

## CONCLUSION

In conclusion, our study is the first to systematically address the prognostic and clinical significance of the NCAPG gene in cancer. We provide meta-analytical and bioinformatic evidence that NCAPG acts as an oncogenic mRNA with great potential as a biological prognostic marker for cancer. However, this study had certain limitations, and more basic experiments are needed to verify these conclusions.

## Supplementary Materials

Supplementary Figures
