# Supplementary material for: NCAPG as a novel prognostic biomarker in numerous cancers: a meta-analysis and bioinformatics analysis
Source: Aging (Albany NY). 2023 Mar 29;15(7):2503–24. doi: 10.18632/aging.204621 (PMC10120898; doi:10.18632/aging.204621)
Supplement: Supplementary Figures [file aging-15-204621-s001.pdf]

SUPPLEMENTARY FIGURES

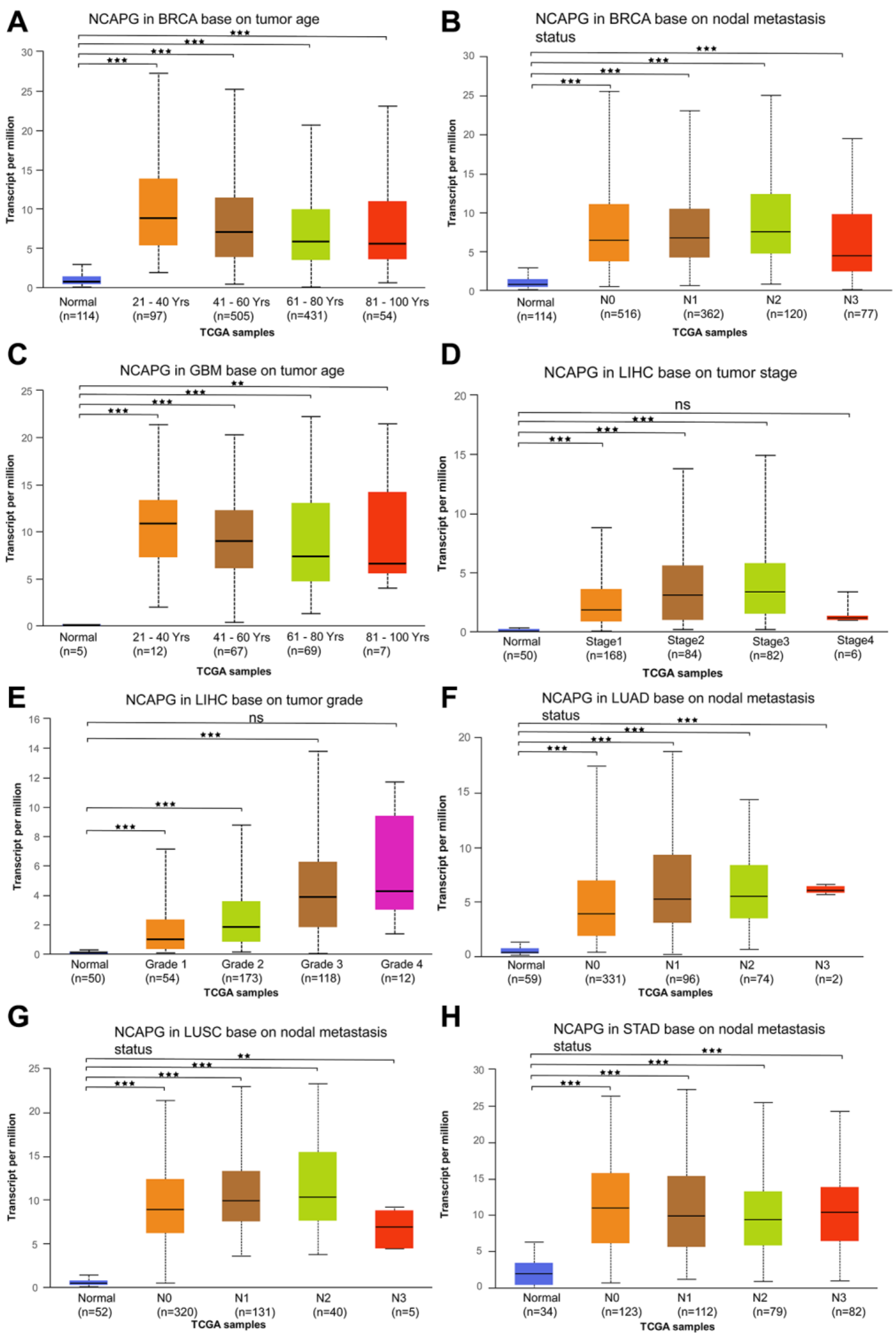

Supplementary Figure 1. NCAPG in BRCA base on tumor age (**A**), NCAPG in BRCA base on nodal metastasis status (**B**), NCAPG in GBM base on tumor age (**C**), NCAPG in LIHC base on tumor age (**D**), NCAPG in LIHC base on tumor grade (**E**), NCAPG in BRCA base on nodal metastasis status (**F**), NCAPG in LUSC base on nodal metastasis status (**G**), NCAPG in STAD base on nodal metastasis status (**H**).

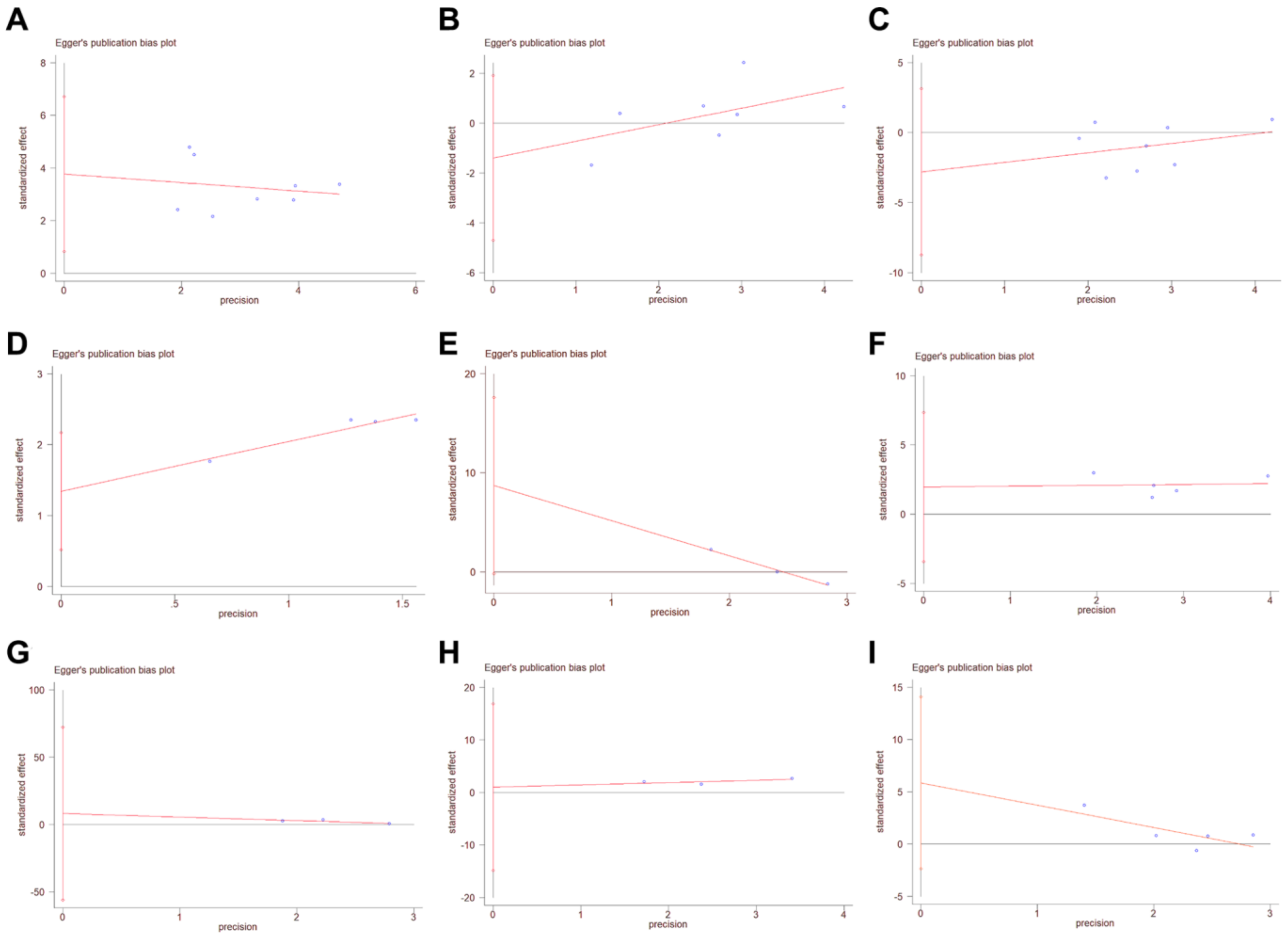

Supplementary Figure 2. Egger's tests for publication bias. (**A**) OS, (**B**) gender, (**C**) age, (**D**) distant metastasis, (**E**) differentiation, (**F**) lymph node metastasis, (**G**) relapse, (**H**) clinical stage, (**I**) T classification.

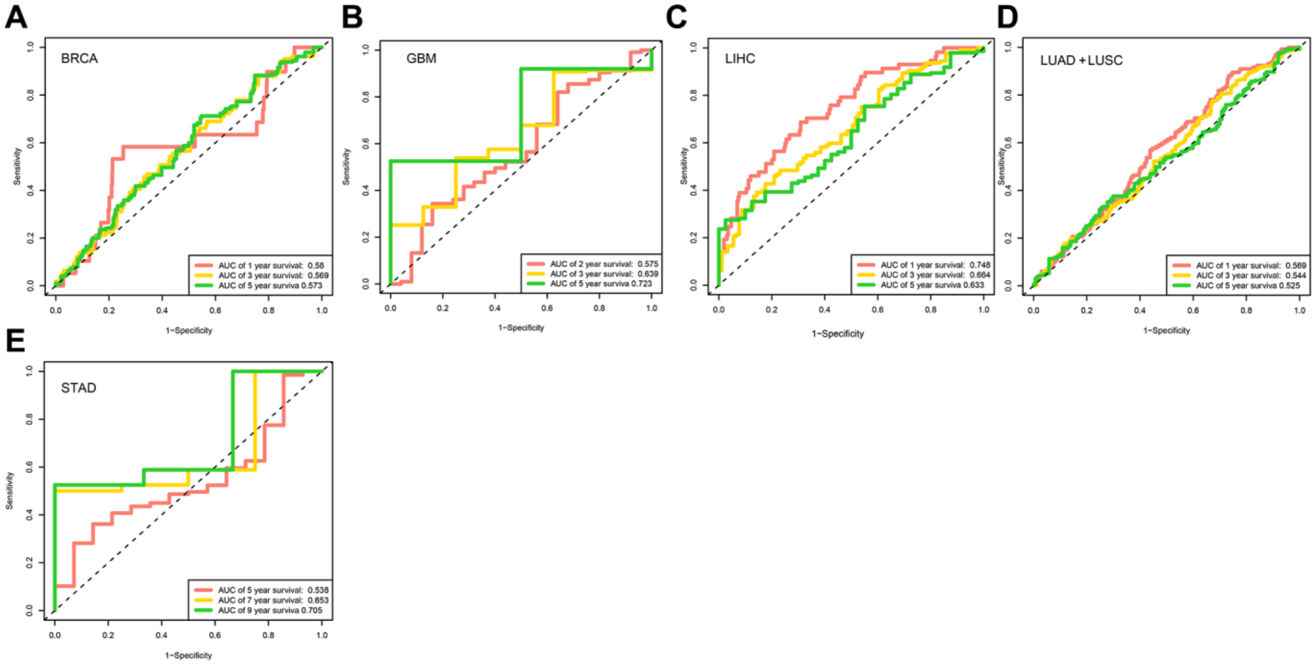

Supplementary Figure 3. (**A**) BRCA's time ROC curve, (**B**) GBM's time ROC curve, (**C**) LIHC's time ROC curve, (**D**) LUAD and LUSC's time ROC curve, (**E**) STAD's time ROC curve.
